# Effect of Short-Term Exposure to Supplemental Vibrotactile Kinesthetic Feedback on Goal-Directed Movements after Stroke: A Proof of Concept Case Series

**DOI:** 10.3390/s21041519

**Published:** 2021-02-22

**Authors:** Giulia Ballardini, Alexis Krueger, Psiche Giannoni, Lucio Marinelli, Maura Casadio, Robert A. Scheidt

**Affiliations:** 1 Department Informatics, Bioengineering, Robotics and Systems Engineering (DIBRIS), University of Genoa, 16126 Genoa, Italy; giulia.ballardini@edu.unige.it (G.B.); psichegi@tin.it (P.G.); Maura.Casadio@unige.it (M.C.); 2 Department Biomedical Engineering, Marquette University and Medical College of Wisconsin, Milwaukee, WI 53233, USA; kruegeralexisr@gmail.com; 3 Department of Neuroscience (DINOGMI), University of Genoa, 16132 Genoa, Italy; lucio.marinelli@unige.it; 4 Division of Clinical Neurophysiology, Department of Neuroscience, IRCCS Ospedale Policlinico San Martino, 16132 Genoa, Italy; 5 Feinberg School of Medicine, Northwestern University, Chicago, IL 60611, USA; 6 Division of Civil, Mechanical and Manufacturing Innovation, National Science Foundation, Alexandria, VA 22314, USA

**Keywords:** reaching, stabilization, biofeedback, haptic feedback, proprioception

## Abstract

Many survivors of stroke have persistent somatosensory deficits on the contralesional side of their body. Non-invasive supplemental feedback of limb movement could enhance the accuracy and efficiency of actions involving the upper extremity, potentially improving quality of life after stroke. In this proof-of-concept study, we evaluated the feasibility and the immediate effects of providing supplemental kinesthetic feedback to stroke survivors, performing goal-directed actions with the contralesional arm. Three survivors of stroke in the chronic stage of recovery participated in experimental sessions wherein they performed reaching and stabilization tasks with the contralesional arm under different combinations of visual and vibrotactile feedback, which was induced on the ipsilesional arm. Movement kinematics were encoded by a vibrotactile feedback interface in two ways: state feedback—an optimal combination of hand position and velocity; and error feedback—the difference between the actual hand position and its instantaneous target. In each session we evaluated the feedback encoding scheme’s immediate objective utility for improving motor performance as well as its perceived usefulness. All three participants improved their stabilization performance using at least one of the feedback encoding schemes within just one experimental session. Two of the participants also improved reaching performance with one or the other of the encoding schemes. Although the observed beneficial effects were modest in each participant, these preliminary findings show that supplemental vibrotactile kinesthetic feedback can be readily interpreted and exploited to improve reaching and object stabilizing actions performed with the contralesional arm after stroke. These short-term training results motivate a longer multisession training study using personalized vibrotactile feedback as a means to improve the accuracy and efficacy of contralesional arm actions after stroke.

## 1. Introduction

About 5 million people survive a stroke each year worldwide [1]. More than 50% of survivors exhibit long-term weakness or paresis in their contralesional arm [2,3], while 50% exhibit deficits of kinesthesia (i.e., the sensation of limb position and movement) [4,5,6]. Many survivors present with deficits of proprioception but still retain the ability to generate movement [6]. In healthy people, movements are typically composed of at least two independent control actions generating movement trajectories and hand stabilizations [7,8]. Many activities of daily living such as reaching for a cup of water or using a spoon to eat soup require both aspects of control. Because movement trajectories are often less accurate and stabilization strategies are less effective after stroke [9], many survivors give up using their contralesional limbs even though this impedes physical interaction with people and things, thereby reducing their quality of life [10].

Traditionally, rehabilitative treatments have focused on motor retraining [11,12], with only limited attention paid to mitigating proprioceptive deficits and negative impacts on sensorimotor performance (see [13] for a review). Not only do proprioceptive deficits interfere with motor learning, but people with proprioceptive impairments have sub-optimal functional recovery [5,14]. Although people suffering loss of proprioceptive feedback can move by relying on vision of their limbs, long processing delays inherent to the visual system (100–200 ms; [15]) yield movements that are typically slow, poorly coordinated, and require great concentration [16,17]. Visually guided corrections come too late and result in jerky, unstable movements [18]. What can be done to improve sensorimotor control after stroke in a way that translates into increased arm use and improved quality of life?

Several research groups have sought to use wearable technologies to enhance upper extremity (UE) control in healthy people by guiding ongoing movement with supplemental kinesthetic cues in various forms: vibrotactile stimuli [19,20,21,22], skin stretch cues [23,24,25], and force feedback [26,27,28]. Providing supplemental kinesthetic cues may also be a plausible way to mitigate the impact of proprioceptive deficits on UE control after stroke. Recently, empirical evidence has demonstrated the potential benefit of vibratory stimuli for improving neurorehabilitation of the arm and hand [29,30,31,32,33,34], for increasing ankle strength and standing stability [35,36], and for restoring gait symmetry during walking [37,38,39]. However, to the best of our knowledge, no studies to date have investigated the use of real-time supplemental vibrotactile kinesthetic feedback into the ongoing control of goal-directed movements and stabilizations of the arm and hand after stroke.

This proof-of-concept pilot study sought to assess the feasibility of using vibrotactile kinesthetic feedback to enhance the accuracy and precision of goal-directed stabilization and reaching tasks performed without visual feedback by survivors of stroke in the chronic stage of recovery. We were motivated by the results of Krueger et al. [40], who showed that neurologically intact individuals could immediately use two forms of supplemental feedback to improve the accuracy and precision of goal-directed stabilization and reaching actions; these included: vibrotactile limb state feedback comprised of an optimal combination of hand position and velocity information encoded in a static external Cartesian frame of reference, and vibrotactile hand position error feedback, which encoded the vector difference between the hand’s instantaneous position and its intended spatial target. Following the approach of Krueger et al. [40], we present three cases demonstrating the extent to which individual stroke survivors with different sensorimotor impairments can interpret and integrate the two forms of supplemental kinesthetic feedback into the real-time control of stabilization and reaching actions performed with the contralesional arm. The subjects participated in at least two experimental sessions on separate days, wherein we evaluated the subjective experience and the immediate objective utility of the two encoding schemes on enhancing the accuracy and precision of stabilization and reaching actions performed with the arm and hand. We tested the hypothesis that, like neurologically intact individuals [40], chronic stroke survivors without cognitive impairment can readily interpret the informative content of vibratory kinesthetic feedback and use it to solve reaching and stabilization tasks. We also examined the hypothesis that performance would be consistently better with an error encoding scheme vs. state encoding, as found previously for neurologically intact subjects [40].

## 2. Materials and Methods

### 2.1. Subjects

A convenience sample of three survivors of stroke in the chronic stage of recovery (aged 57 to 68 years; 2 females; see Table 1 for details) provided written and informed consent to participate in this study. The study was comprised of experimental sessions designed to evaluate the immediate utility and usability of supplemental kinesthetic feedback for enhancing the control of stabilization and reaching movements of the arm and hand. Participant inclusion criteria included: (i) diagnosis of a single stroke event confirmed by brain imaging; (ii) within the chronic stage of recovery (i.e., more than six months post-stroke); (iii) capability to perform upper limb movement exceeding 10 cm in presence of counterbalance support; (iv) capability of understanding and following basic two-step instructions: a Folstein Mini-Mental State Examination score above 28 [41]; and (v) normal or corrected-to-normal vision. Exclusion criteria included: (i) absence of vibration sensation in the ipsilesional arm; (ii) neurological impairments that prohibit informed consent and the understanding of the tasks; and (iii) presence of hemi spatial neglect. All subjects were enrolled by a qualified physiotherapist and a neurologist. All procedures were approved by a local Ethical Committee serving the University of Genoa (ASL3 Genovese) and the Institutional Review Board of Marquette University (HR-3044) in accord with the 1964 Declaration of Helsinki. The study required one visit to the lab for clinical testing and then two separate 1-h experimental sessions, all performed within a three-week period.

### 2.2. Clinical Evaluations

A licensed physiotherapist evaluated the motor, functional and proprioceptive status of each subject using a series of clinical assessments (Table 2). These included: (i) the Upper Extremity portion of the Fugl-Meyer Assessment (FMA-UE), which includes tests of motor impairment and somatosensation in the contralesional arm. Higher FMA-UE scores indicate less impairment; (ii) the Modified Ashworth Scale (MAS) to quantify stiffness; higher scores of MAS indicate more spasticity; (iii) the 13-item Chedoke Arm and Hand Activity Inventory (CAHAI), which is a test of sensorimotor function. Higher CAHAI scores mean better functional ability in activities of daily living; (iv) the kinesthetic and stereognosis portions of the Nottingham Sensory Assessment (NSA) in the contralesional arm. Higher scores indicate better somatosensory capability; (v) a tuning fork assessment of vibrotactile sensation in both arms. Higher scores indicate better sensation.

### 2.3. Experimental Set-Up

**Figure 1:**
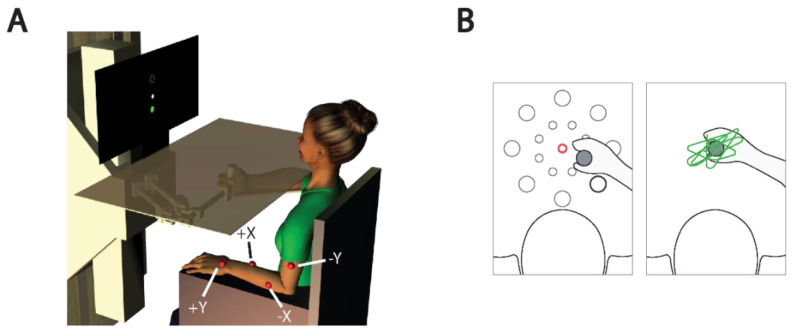
Experimental set-up and tasks. (**A**) Subject seated in a high-backed chair holding the end effector of a planar manipulandum with the contralesional hand. An opaque screen prevented direct visual feedback of the moving arm and end effector. The four actuator vibrotactile interface was fixed to the stationary ipsilesional arm. The default locations of the vibration motors are shown by red spheres. Each motor was activated by contralesional hand displacement in one of the four cardinal directions: {+x, −x, +y, −y}. A vertical screen was placed in front of the subject to provide visual cues of target position, and in some conditions, visual feedback of hand motion. (**B**) Tasks—Left: example of a reaching movement from the starting target (black) to the final target (red). All possible target locations are shown here in gray. Right: example of hand stabilization against robotic perturbations at the center of the robot workspace. Hand displacements are shown in green.

Subjects were seated comfortably in a high-backed chair with a flat footrest in front of a horizontal planar robotic manipulandum (Figure 1A; see [42] for a detailed description). The contralesional hand grasped the robotic handle, which has an integrated light-weight and rigid arm support that was strapped to the forearm. The arm support provided gravity compensation and free motion of the forearm in the horizontal plane. The ipsilesional arm rested comfortably on a horizontal support mounted below the robot’s plane of motion. An opaque shield was placed over the workspace to block the subject’s view of the moving arm and the robotic apparatus. The chair was adjusted to align the left/right horizontal center of the robot’s workspace with the subject’s midline. The subject was positioned near the edge of the opaque shield, so the anterior/posterior range of the robot was within the subject’s reach. The seat height was adjusted such that the abduction angle of the shoulder was between 75° and 85°. A vertical screen was placed in direct view, 70 cm from the subject; it always provided visual cues of target position and hand motion when appropriate (see Experimental Protocol section, below). The spatial mapping from handle movement to cursor movement was 1:1. Before starting each experimental session, subjects were provided descriptions of the tasks they would be asked to perform and encouraged to ask questions. Subjects were also encouraged to give verbal feedback about the ongoing experience at any time during the experimental sessions.

### 2.4. Tasks

We focused on two actions that are fundamental to the performance of many activities of daily living: reaching and stabilizing with the arm and hand [7,16,43].

#### 2.4.1. Reaching

In each block of the reaching task, subjects were asked to perform center-out-and-then-back reaches to 16 targets, for a total of 32 discrete reaching movements. Each movement was considered as a unique trial. The 16 targets were equally distributed around two concentric circles centered on the center of the robot’s workspace (Figure 1B, left). This design allows testing of 16 movement directions (22.5° apart) and two movement extents: 5 and 10 cm for the eight targets fixed to the inner and the outer circles, respectively. To equalize the apparent difficulty of reaching targets placed at different distances, we scaled the target size in visual cues according to Fitts’ Law [44] such that the target radius was 1 cm for the close targets and 2 cm for the far targets. Target presentation order was pseudo-randomized within each block. Subjects were instructed to “Capture the target as quickly and accurately as possible.” As a reminder to capture the target quickly, reach targets turned from red to blue 1 s after they appeared. Upon completing the reach, the participant announced that they thought they had arrived at the target and the experimenter registered that event (i.e., the end of the movements) by pressing a button. Subjects were allowed a maximum of 20 s to complete each trial.

#### 2.4.2. Stabilizing

In each block of the stabilization task, subjects attempted to hold the robot handle steady at the center of the workspace for 60 s against time-varying sum-of sinusoid force perturbations (Figure 1B, right). The perturbations contained both a low frequency and several high frequency components (Equation (1a) and (1b)):

(1a) $$Fx=0.75\cdot \cos\left(2\pi \cdot 1.75t\right)+\;0.75\cdot \cos\left(2\pi \cdot 1.2t\right)+\;6\cdot \cos\left(2\pi \cdot 0.25t\right)$$

(1b) $$Fy=0.75\cdot \sin\left(2\pi \cdot 1.65t\right)+\;0.75\cdot \sin\left(2\pi \cdot 1.1t\right)+\;6\cdot \sin\left(2\pi \cdot 0.25t\right)$$

Accordingly, hand force perturbations had peak magnitudes of approximately 10 N.

### 2.5. Vibrotactile Interface

Supplemental kinesthetic feedback about the moving hand was provided using a two-channel (four-actuator) vibrotactile interface attached to the non-moving ipsilesional arm. Each actuator was an eccentric rotating mass (ERM) micromotor with an operational frequency range of 50–250 Hz (Pico Vibe 310-117; Precision Microdrives, Inc.; London, UK). The ERM actuators have a vibrational amplitude range that is 0.20–0.97 N and covaries with vibrational frequency (see [40] for more details). The fact that vibration frequency and amplitude are coupled in ERM actuators is well-suited for the purpose of implementing a vibrotactile interface because as shown previously by Cipriani et al. [45], people perceive vibrotactile stimuli better when the amplitude and frequency of vibration increase or decrease coherently. For simplicity in the text to follow, we will refer to correlated changes in the amplitude and frequency of vibrotactile stimuli as changes in vibration intensity.

The actuators were initially arranged with a standard configuration designed such that inter-actuator spacing exceeded two-point discrimination thresholds for dermatomal regions of the arm and forearm as reported by Nolan [46]. In the standard configuration, the actuators—represented in Figure 1A by red spheres—were placed at least 6 cm apart [45]. One actuator (Y+) was placed on the back of the hand approximately 1 cm proximal to the first and second finger metacarpophalangeal joints. Two actuators were placed on the forearm between 3 to 7 cm distal to the cubital fossa, one on each side of the forearm (X− on the left, X+ on the right with respect to the subject’s reference frame). One actuator (Y−) was placed on the biceps muscle belly about 5 cm proximal to the cubital fossa. Each actuator was secured by an elastic band. The actuators were used to encode hand motion into vibratory stimuli as a vector, with each dimension of Cartesian space mapped onto one pair of the actuators.

Prior to each experimental session, we performed a set-up procedure for the vibrotactile interface that lasted approximately 5–10 min: We adjusted the actuator locations if necessary, so that the subject could indicate reliably which actuator or pair of actuators was activated at any given time. The vibration of all actuators was zero at the center of the workspace. We assessed the ability of subjects to correctly perceive vibratory stimuli with the hand at three distances from this point corresponding to low, middle, and high intensity vibrations (approximately 10%, 40% and 90% Full Scale Range (FSR: 75–250 Hz), respectively). Set-up began with the subject placing the cursor at each of the four corners of the screen (corresponding to displacements of 15 cm from the center), and then reporting which actuators were vibrating (at ~90% FSR). This was repeated two additional times, once near the center of the screen (~10% FSR) and once approximately mid-way between the center and the edge of the screen (~40% FSR). Next, the subject was asked to place the cursor at the center of the screen, and then, to move away from that location and back again in each cardinal direction. During setup, one subject could not give a clear and correct indication for two actuators as to which actuator was active and/or the appropriate direction of intensity change in that actuator. We adjusted these actuator locations to the nearest point where the vibrotactile stimuli were correctly perceived, always maintaining a minimum distance of 6 cm between actuators [45]. In each case, the actuator was moved no more than 3 cm from its default location.

### 2.6. Kinesthetic Feedback Encoding Schemes

Subjects experienced two different forms of supplemental kinesthetic feedback during the experiments: a vibrotactile encoding of limb state feedback, and an encoding of hand position error feedback. Both types of feedback conveyed meaningful information about the subject’s performance in that the vibration encoded the motion of the hand with respect to either the center of the workspace (state feedback) or the current target (error feedback). In both cases, motion with respect to the reference point in the rightward/leftward and forward/backward directions resulted in vibrations of the +X/−X and the +Y/−Y actuators, respectively.

#### 2.6.1. State Feedback

In this encoding scheme, the intensity of vibration was a weighted linear combination of hand position and velocity information as per Krueger et al. [40] (Equation (2)):

(2) $$\gamma \left(t\right)=0.2\cdot \dot{p}\left(t\right)+0.8\cdot p\left(t\right)$$

where, $p\left(t\right)$ and $\dot{p}\left(t\right)$ represent hand position and velocity vectors in extrinsic coordinates, and $\gamma \left(t\right)$ represents the vector of vibration intensity that is mapped into the four-actuator vibrotactile interface as a function of time. The sign and the value of each element of $\gamma \left(t\right)$ determined which actuator was turned on and with what intensity. As described in Section 2.5 (Vibrotactile interface), each actuator encoded a hand displacement along one of the cardinal directions {+X, −X, +Y, −Y} relative to the center of the hand’s workspace. The particular weighting of position and velocity information of Equation (2) was found to yield an optimal performance during reaching and stabilizing tasks performed by neurologically intact individuals [40]. The center of the vibrotactile workspace (i.e., the point where the vibration of all actuators was zero if the hand was held in that position) was aligned with both the center of the visual screen and the center of the robot’s workspace. Vibratory stimulation reached 90% FSR when the hand was held at the bounds of the visual display, 60% FSR at the far targets, and 30% FSR at the close targets.

#### 2.6.2. Error Feedback

Here, vibratory stimuli encoded information about the signed error between the hand’s instantaneous location and the current target’s location. The vibration was zero when the hand was at the center of the current target, and its intensity increased in proportion to the Euclidean distance from that target. Vibratory stimulation reached 90%, 60% and 30% full scale range when the hand was 15, 10, and 5 cm respectively from the then-current target. With error feedback, the vibratory stimuli conveyed no information about hand velocity. Error feedback provided information only about hand position relative to the target, which changed from one trial to the next in the reaching task.

### 2.7. Experimental Protocol

Each subject participated in two experimental sessions on separate days. Each session lasted up to 90 min. One subject volunteered to participate in a third session, which assessed the possibility of day-over-day performance improvements (i.e., sensorimotor learning) in the integration of supplemental vibrotactile kinesthetic feedback into the control of reaching movements after stroke. All sessions were performed within three weeks of the clinical evaluation. During each session, we provided only one type of vibrotactile feedback (state or error). In the first session all subjects experienced state feedback, whereas all subjects experienced error feedback in the second (and later) session(s). In each session, the subjects performed several blocks of trials in three experimental phases, each with different visual feedback conditions and purposes. These phases included familiarization, practice, and assessment. Each phase was composed of one or more trial blocks wherein subjects performed stabilization and/or reaching tasks under a specific combination of vibroTactile (T) and Visual (V) feedback. The protocol performed by each subject varied in the number of blocks performed within each phase due to differing levels of stamina between subjects and across testing sessions (see Table 3 for details).

#### 2.7.1. Familiarization (V+T−)

In the familiarization phase, subjects completed the reaching and the stabilization tasks without vibrotactile feedback (T−). They were provided visual feedback of hand position (V+) through a 0.5 cm radius cursor that was continuously visible on the computer screen. This block was performed in the first experimental session; it was intended to ensure that subjects understood the two tasks. While the familiarization phase was also offered to all subjects at the beginning of the later session(s), all three of them declined, stating that they understood the reaching and stabilization tasks and were comfortable repeating them without further practice.

#### 2.7.2. Practice (V_KR_T+)

In the practice phase, subjects performed at least two blocks of the reaching task with the vibrotactile feedback always on (T+). The practice phase did not include the stabilization task. Real-time visual feedback of hand position was provided on the screen only after the end of each trial (i.e., Knowledge of Results (KR); V_KR_). Subjects were encouraged to use the terminal visual feedback to correct any target capture error that may have accrued during the initial reach. The goal of this phase was to encourage subjects to learn the mapping between hand position and the information encoded in the vibrotactile feedback.

#### 2.7.3. Assessment (V−)

After practice, subjects underwent an assessment phase, wherein they performed the reaching and stabilization tasks without any visual feedback (V−) during or after each reach. The cursor representing hand position/motion was never displayed on the screen, knowledge of results was not provided, and actual vision of the hand was precluded by the opaque shield (see Figure 1A). This phase was divided in two separate blocks, one with vibrotactile feedback and the other without. In the first block (Baseline), the subjects did not receive any external visual or vibrotactile feedback (V−T−). The goal of this block was to assess the baseline capability of the subjects to complete the tasks using only their residual inherent proprioception. In the second block (Generalization), the vibrotactile feedback was turned back on (V−T+). The goal of this block was to test the subject’s ability to generalize what they learned during practice with vibrotactile feedback (state or error) and visual KR to a condition entirely devoid of visual feedback.

### 2.8. Subjective Self-Report Evaluation of the Vibratory Stimuli

At the end of each experimental session, we asked subjects to verbally report on their subjective experience by asking three open-ended questions. We focused specifically on aspects of usability, and user satisfaction. To assess usability, we asked “How easy was it to perceive changes in the vibrotactile signals?” and “How easy was it to use those cues to achieve the goals in each task?”. To assess user satisfaction, we asked “To what extent was the vibrotactile feedback system comfortable to use?”.

### 2.9. Data Analysis

Hand position data were recorded at 1 kHz. The resulting data were subsequently filtered with a zero-phase fourth-order Butterworth low-pass filter with a cut-off frequency of 12 Hz. We computed the following performance measures for each trial for each subject under the two vibrotactile encoding schemes. In the reaching task, we computed the final position error as the Euclidean distance between final hand position and the center of the current target. The final hand position was taken as the hand’s location either when the subject indicated that the target had been reached or when the time for completing the trial had expired, whichever came first. In the stabilization task, we computed the root-mean-square-error (RMSE) to assess how well subjects could maintain the hand at the desired target. To compute RMSE, we discarded the first 10 s of each 60-s trial to eliminate potential start-up transients caused by the onset of hand force perturbations (cf., [40]). We then divided the trial into five non-overlapping 10-s segments and computed the RMSE between the hand’s instantaneous location and the stabilization target (i.e., the center of the workspace). We evaluated the trial RMSE using the mean and standard error values computed from the RMSE values obtained in the five 10-s segments.

We used a single-subject-design analyses to evaluate changes in task performance due to the presence of the vibrotactile feedback. Our primary focus was on the assessment blocks performed without visual feedback. We investigated differences in performance between trials with and without supplemental vibrotactile feedback (i.e., between the Generalization and Baseline blocks), and between Generalization blocks with different vibrotactile feedback encoding schemes (i.e., error vs. state feedback). Secondarily, we focused on the practice blocks, to investigate learning effects as subjects practiced reaching with vibrotactile feedback within and across days.

## 3. Results

### 3.1. Subjective Evaluations of Supplemental Vibrotactile Feedback

#### 3.1.1. User Satisfaction

All three subjects tolerated the vibratory stimuli with no complaints of hypersensitivity or discomfort. When we asked subjects to report on the extent to which the vibrotactile feedback system was comfortable to use, all three stated that using the supplemental vibrotactile feedback to guide the arm was overall a mild positive experience. One subject (S03) did report mild annoyance when vibrations were at their highest intensity levels, saying that the vibrations felt like “a bright light or a loud noise” and that they were a little “distracting”.

#### 3.1.2. Usability

All three subjects reported that they were able to perceive the vibrotactile feedback applied to the ipsilesional arm. For two of the subjects, vibration perception was satisfactory with the actuators placed in their default locations. One of the subjects (S01) experienced initial difficulty perceiving vibrations on the external forearm and on the upper arm. We therefore adjusted the position of these actuators by moving them approximately two centimeters in different directions until the subject could reliably perceive changes in vibration intensity. We also adjusted the elastic band on the internal forearm actuator to increase the applied pressure so as to allow this subject to more effectively perceive the vibration stimuli.

When we asked how easy it was to perceive changes in the vibrotactile signals, all of them responded that error feedback was easier to understand and use than state feedback. S02 remarked that his vibrotactile sensitivity improved with practice, whereas the other two subjects reported a modest perceived degradation in vibrotactile sensitivity after approximately one hour of continuous practice. S02 and S03 reported an increase in alertness or general body awareness while using the supplemental vibrotactile feedback, and that this effect persisted for some time after the experimental session was over. However, these same two subjects also reported difficulty in dividing attention between “feeling” the vibration on the one limb and executing movements with the other. S01 and S03 both expressed difficulty in integrating simultaneous visual and vibrotactile inputs, as occurred in between trials in the Practice blocks (*V_KR_T+*).

### 3.2. General Observations on Kinematic Performance with and without Ongoing Visual Feedback

**Figure 2:**
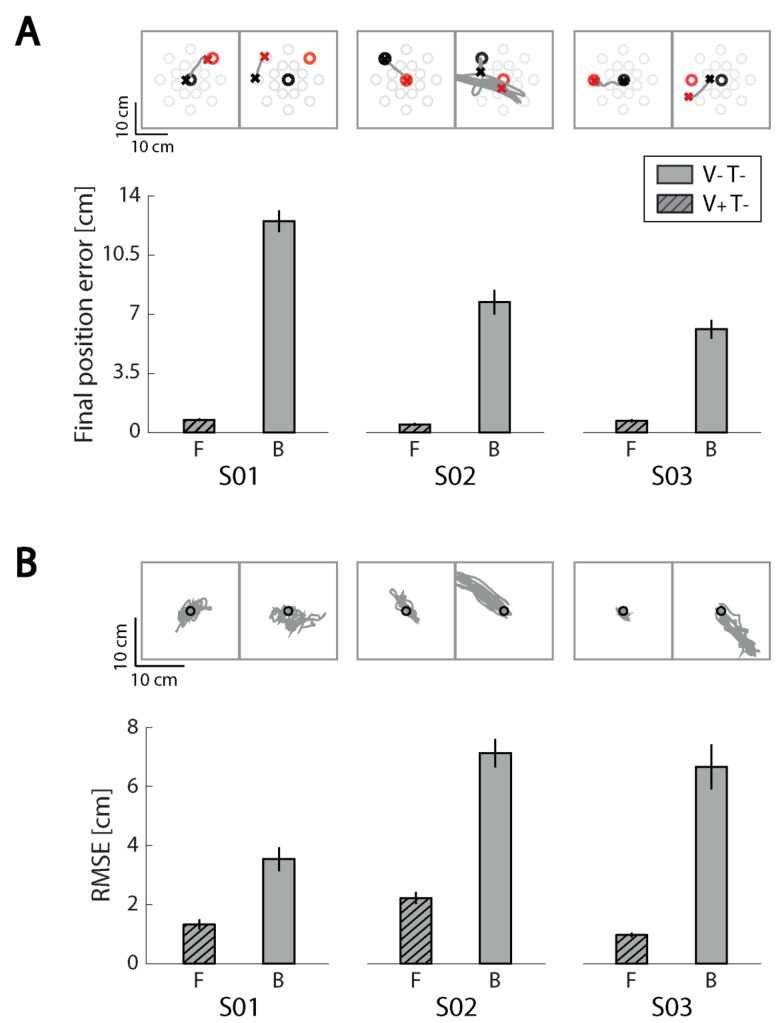
Familiarization and baseline phase performance for the reaching and stabilizing tasks for each subject. (**A**) Reaching task performance. Top row: examples of hand paths for the Familiarization block (F; left) performed with only visual feedback and the Baseline block (**B**; right) performed in the absence of extrinsic feedback. Results for each subject are presented in separate columns. Start and stop targets are represented by black and red ‘o’ symbols, respectively. The start and stop positions of the corresponding hand movements are represented by ‘x’ symbols. Bar charts on bottom row: Final position error averaged within the familiarization and baseline blocks. Error bars: mean ± 1 SEM. (**B**) Stabilization task performance. Top row: black circle is the center of the workspace where the hand should be stabilized. Hand paths during the stabilization period are shown in grey. Bottom row: root-mean-square-error (RMSE) averaged across consecutive 10 s stabilization intervals. Results for each subject are presented in separate columns.

All three subjects demonstrated sufficient motor capability to perform the reaching task with small target capture errors (i.e., with final position error less than 1 cm) and with stereotypically straight hand paths when they were provided visual feedback of ongoing performance (Figure 2A; Familiarization phase, F: V+T−). All three were also able to stabilize their hand with small positioning errors when provided visual feedback of ongoing performance (Figure 2B; F: V+T−); despite the force perturbations in the stabilization task, average RMSE values did not exceed 2.5 cm when ongoing cursor feedback was provided. By contrast, kinematic performance degraded dramatically during both reaching and stabilizing when all extrinsic feedback was eliminated (Figure 2; Baseline assessment, B: V−T−). As we will show, the subjects exhibited varied levels of success when interpreting and using supplemental state- and error-feedback for closed-loop control of the contralesional arm. No systematic improvements in baseline performance without visual feedback were observed from one day to the next.

### 3.3. Effects of Supplemental Kinesthetic Feedback on Performance of Reaching and Stabilizing Tasks

All subjects learned to interpret and use at least one of the vibration feedback encodings to successfully perform the reaching and/or stabilizing tasks. Some were able to use the vibrotactile information to control the arm more readily, whereas others required more time and practice to do so. There were striking differences between subjects regarding the effects of supplemental kinesthetic feedback on task performance and the effects of practice using the supplemental feedback. We therefore describe the pattern of results separately for each case in the paragraphs to follow.

#### 3.3.1. Subject 1 (S01)

**Figure 3:**
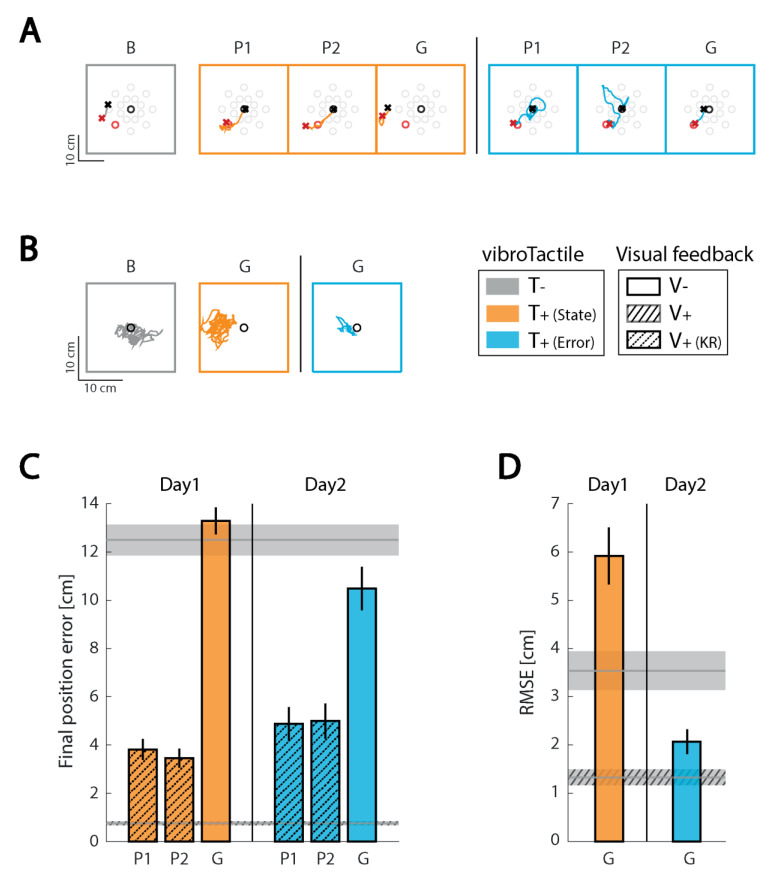
Individual results for subject 1 (S01). (**A**) example hand paths for the Baseline (B), Practice (P), and Generalization (G) blocks in the reaching task. (**B**) example hand paths for the Baseline (B) and Generalization (G) blocks in the stabilization task. Orange: state feedback condition; Blue: error feedback condition; Gray: no vibrotactile or vision feedback. (**C**) Mean (±1 SEM) final position error for the reaching task; (**D**) Mean (±1 SEM) root-mean-square-error (RMSE) for the stabilization task. As before, color coding indicates the type of vibrotactile feedback provided. Upper horizontal gray patch: mean ± 1 SEM of performance in the Baseline block of trials without visual or vibrotactile feedback. Lower (dashed) horizontal gray patch: mean ± 1 SEM of performance in the familiarization block of trials (i.e., with concurrent visual feedback).

*Reaching.* During baseline assessment (i.e., in the absence of all extrinsic feedback), S01 performed inaccurate reaches that were generally shorter than those required to perform the cued task. These movements were also shifted relative to the intended start and goal targets (Figure 3A Baseline, B). This result is consistent with previous observations of “proprioceptive drift” [47], which is thought to arise due to an accumulating misalignment of visual and proprioceptive representations of limb position. Adding visual KR to either form of real-time supplemental vibrotactile feedback mitigated the drift effect to a large extent, primarily by shifting the initial hand position back to the desired starting location (Figure 3A; Practice, P). This mitigation was evidently due to the visual KR and not to the presence of supplemental kinesthetic feedback because drift in the hand’s initial position re-established rapidly when only vibrotactile feedback was provided (Figure 3A; Generalization, G). These single-trial observations were consistent within each testing day (i.e., for both encoding schemes of vibrotactile feedback; Figure 3C). Note that this subject decreased final position error in the generalization blocks by 16.1% with error feedback relative to her baseline trials (which are represented by the upper grey horizontal band in Figure 3C), whereas final position errors increased by 6.3% with state feedback relative to baseline performance.

*Stabilization.*Figure 3B shows individual stabilization trials for each phase in the two experimental sessions. Differences in generalization block performance between the two vibrotactile feedback encodings were more dramatic in stabilization than in reaching. Hand deflections were smaller in magnitude and less shifted with respect to the center of the workspace when this subject stabilized the hand with error feedback as compared to state feedback. These observations were reflected in the RMSE values, which decreased by 41.6% with error feedback relative to baseline and increased by 67.2% in the state feedback test block relative to baseline (Figure 3D). The increase in RMSE with state feedback testing was largely due to a reappearance of hand positioning errors accruing in the absence of visual feedback.

In summary, S01 was able to interpret and use vibrotactile error feedback to enhance closed-loop control of contralesional arm reaching and stabilization actions in just one experimental session. By contrast, limb state feedback did not as rapidly enable improved performance in the absence of visual feedback on either task relative to V−T− baseline trials.

#### 3.3.2. Subject 2 (S02)

**Figure 4:**
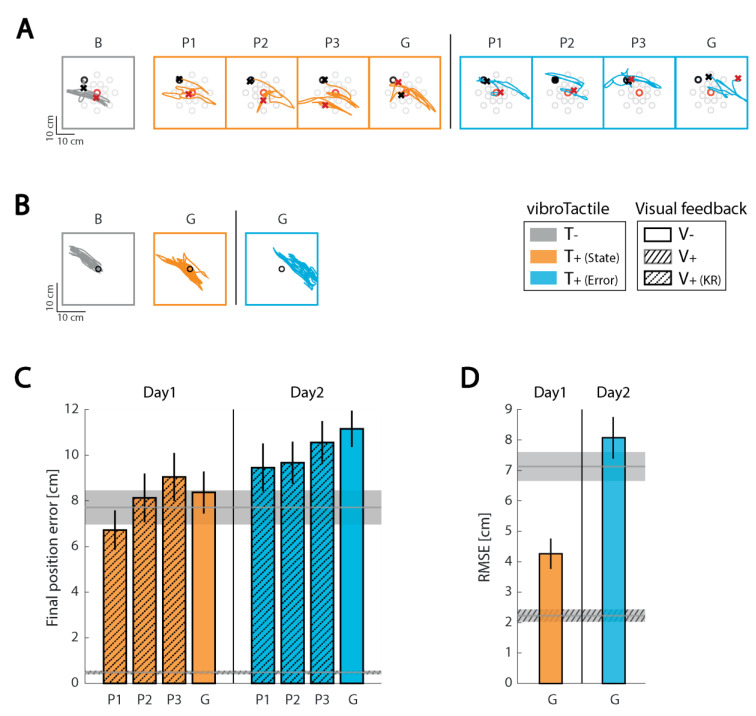
Individual results for subject 2 (S02). Panels and presentation as in Figure 3.

*Reaching.* S02 persistently made multiple corrective movements when reaching in the absence of concurrent visual feedback—with or without supplemental kinesthetic feedback. Reach performance degraded substantially when vision was removed, and this subject was unable to capitalize on either form of supplemental kinesthetic feedback to reduce target capture errors (Figure 4A). Even adding visual KR in the practice blocks failed to mitigate the performance degradation within a single session of practice with either kinesthetic encoding scheme. Error feedback appeared to confound this subject more than state feedback during reaching (Figure 4C).

*Stabilization.* Performance also degraded in the stabilization task during baseline assessment without concurrent visual feedback; hand deflections became larger and displaced relative to the center of the workspace (Figure 4B). In contrast to this subject’s performance in the reaching task, stabilization improved markedly using state vibrotactile feedback (a 40.2% reduction in RMSE relative to the no-feedback baseline trial), mainly by reducing hand position drift (Figure 4D, Day 1). By contrast, error feedback led to a 13.2% increase in RMSE relative to baseline trials. While both encodings convey information primarily about the hand’s position relative to the center of workspace in this task, only state feedback includes velocity information that accentuates changes in hand position, which may have helped this subject perform a more effective error correction when stabilizing.

Thus, while S02 was able to exploit supplemental limb state feedback to improve stabilization of the contralesional arm, he was unable to use error feedback effectively in that task. S02 was unable to use either encoding scheme to improve performance in the reaching task.

#### 3.3.3. Subject 3 (S03)

When S03 experienced state feedback during the first experimental session, she tried to nullify the vibratory stimulation as if she were receiving error feedback. This behavior was persistent; even after repeated explicit instructions on how to use state feedback, S03 declared that state feedback was confusing, that it required high cognitive effort, and that she preferred not to continue using state feedback. The experience did not dampen S03′s willingness to participate in the study because she agreed to perform the second experimental session with error feedback and she also volunteered to attend a third session. The Day 3 session repeated the Day 2 protocol using error feedback.

**Figure 5:**
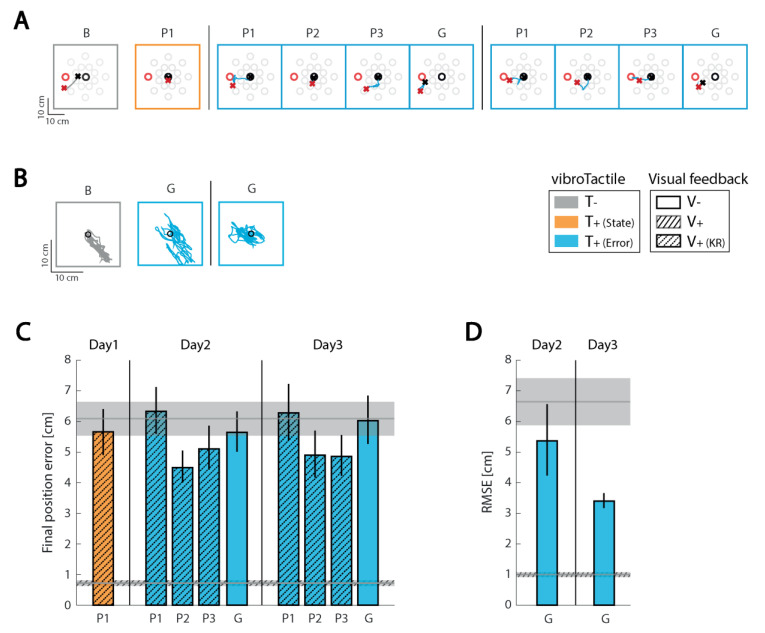
Individual results for subject 3 (S03). Panels and presentation as in Figure 3.

*Reaching.* As for the other two subjects, removing continuous visual feedback strongly degraded S03′s reaching performance, resulting in longer and shifted hand paths relative to the desired start and final positions. With state feedback, S03 hardly moved from the starting point in the first experimental session (Figure 5A), clustering most of the final hand positions close to the center, i.e., where the vibration was absent. When presented on Days 2 and 3 with supplementary error feedback and concurrent visual KR, this subject improved reach performance within each experimental session. The final position error in the third practice block was lower by 19.2% with respect to the first practice block on Day 2, and by 22.7% on Day 3 (Figure 5C). In the last practice block on Day 3, the final position error averaged 20.2% lower than during baseline assessment. Any beneficial effect of practice was likely due to the presence of terminal visual KR for this subject because removing visual KR during the generalization trials effectively eliminated the positive training effect observed during the practice blocks. Performance in the generalization assessment block did not differ from baseline (with final position errors being only 7.4% and 1.1% lower in the generalization assessments of Day 2 and 3, respectively). We observed no clear day-over-day improvements in reach performance during practice with error feedback in this subject.

*Stabilization.* In the stabilization task—as in reaching—this subject relied heavily on visual feedback. During baseline assessment (i.e., in the absence of all extrinsic feedback), hand deflections became larger with respect to the familiarization trial block and displaced relative to the central target (Figure 5B(B)). The application of supplementary error feedback partially mitigated this effect, leading to a lower RMSE in the generalization block (19.1% lower than in the baseline block; Figure 5D). With repeated practice using error feedback (i.e., on Day 3), the hand’s position was much better stabilized on the workspace center, leading to a RMSE 48.8% lower than in the baseline trials, reflecting a substantial day-over-day learning effect.

In summary, while S03 was confounded by supplemental limb state feedback, she was able to properly interpret error feedback and use it to improve arm stabilization performance to a modest extent after a single day’s training, and to a larger extent after two days of training.

## 4. Discussion

This proof-of-concept pilot study evaluated the ability of three stroke survivors to interpret and use supplemental vibrotactile kinesthetic feedback to enhance the accuracy and precision of stabilization and reaching actions performed with the contralesional arm and hand in the absence of visual feedback. The supplemental kinesthetic feedback had objective utility in the sense that after only minutes of practice, each of the subjects was able to interpret and use vibrotactile cues to stabilize the hand against unpredictable force perturbations. The subjects differed, however, with regards to which form of information encoding enhanced performance in that task: one of the subjects performed best when vibrations encoded limb state information, whereas the other two performed better when vibrations encoded hand position errors. Only one subject demonstrated the ability to interpret and use error feedback to improve the accuracy of reaches performed without visual feedback; none of them successfully used state feedback to improve reach accuracy within the short one-hour time frame of a single experimental session. Nevertheless, all three subjects reported that using supplemental vibrotactile kinesthetic feedback yielded a positive user experience. When asked to compare the two encoding schemes, all three subjects reported that error feedback was easier than state feedback to understand and use. Improvement of stabilization performance across repeated sessions in one subject suggests that the integration of supplemental kinesthetic feedback into the ongoing control of the arm is a skill that can be learned with practice. Taken together, these results demonstrate that a wearable system providing supplemental kinesthetic feedback can have objective utility for enhancing the control of reaching and stabilizing actions performed with the arm and hand after stroke, while also providing a favorable user experience.

### 4.1. Human Performance Enhancement through Vibrotactile Cueing

A growing body of research has sought to use vibrotactile stimuli to enhance human performance in healthy individuals (e.g., [48,49]) or to overcome sensorimotor deficits in patients (e.g., [50,51,52]). In some cases, uninformative “noisy” stimuli have been used to enhance somatosensory sensitivity to faint stimuli through stochastic resonance [53] or to improve motor coordination by enhancing cortical modulation of spinal reflex activity (c.f., [32,54]). In other cases, important aspects of task performance were encoded into vibrotactile “alerts” intended to increase the user’s situational awareness [49,55,56], or into a continuous stream of vibrotactile cues intended to either teach desirable skills that should persist after the vibrotactile stimuli are removed [19,50,57,58] or to enhance sensorimotor performance through permanent feedback devices designed to be used indefinitely like a prosthesis ([59]; see also [21,22,40]). The system tested in the present study is of the last type in that it is intended to be worn continuously as a real-time sensorimotor control aid, and to provide continuous benefit while worn. We found that after only minutes of practice, all three stroke survivors were able to interpret and use vibrotactile cues to enhance modestly the control of UE reaching and/or stabilizing actions in the absence of concurrent visual feedback. How is this possible?

A recent review of sensory augmentation applied to human balance control highlights four potential mechanisms of action [60], which we now consider as potential means by which supplemental vibrotactile kinesthetic feedback might have enhanced control of UE reaching and stabilization in our study. A first possibility, “sensory restoration”, implies the full restoration of missing sensory information [60]. In the case of UE reaching and stabilizing, this would require the restoration of proprioceptive feedback pathways serving muscle spindle primary (Ia) and secondary (II) afferents, Golgi tendon organs, and the various cutaneous mechanoreceptors (cf. [61]). While our limb state feedback encoding was inspired by the biological encoding of displacement and rate-of-displacement information by muscle spindle primary afferents [40], the application of vibrotactile stimuli in our studies is optimized to preferentially excite Pacinian corpuscles [22,40] rather than directly engaging muscle spindles, tendon organs and their afferent pathways. We do not suggest that we somehow reactivate injured somatosensory feedback pathways serving the contralesional arm through the application of supplemental vibrotactile feedback to the ipsilesional arm. The effectiveness of our approach was not driven by sensory restoration.

A second possibility, “sensory integration”, refers to the optimization of sensorimotor control through a guided re-weighting of intact afferent signal pathways [60]. Exposure to supplemental kinesthetic feedback on the ipsilesional limb during performance of specific actions would provide the central nervous system (CNS) with task-related vibrotactile stimuli that are strongly correlated with residual (intact) afferent signals from the contralesional limb. Repeated success on tasks performed with supplemental kinesthetic stimuli would promote increased weighting of the intact sensory channels, thereby promoting increased reliance on residual intrinsic pathways during performance of the practiced tasks, and possibly during performance of unpracticed tasks. It is expected therefore that training with sensory augmentation would lead to beneficial changes in sensory integration that are maintained even without continued use of the sensory prosthesis [60]. We do not believe that sensory integration contributed significantly to the effectiveness of supplemental kinesthetic feedback in our current study because subjects demonstrated enhanced performance after just a few minutes of practice. This does not seem to be a sufficient amount of time to drive substantially increased reliance on residual afferent signals. Moreover, we did not observe systematic improvements in baseline performance from one day to the next, as would be expected if short bouts of training with the supplemental kinesthetic feedback had led to greater reliance on residual (intact) proprioceptive afferent signals.

A third possibility, “sensory substitution”, refers to synthesis and delivery of artificial motion information replacing that of a damaged source [60]. The idea here is to circumvent injured sensorimotor feedback pathways by encoding motion information into stimuli that the CNS is able to integrate into the implicit planning and control of action. Ideally, the supplemental stimulus encoding would sufficiently replicate the information lost due to injury such that the CNS would draw upon the supplemental information source instead. Likely, however, is the case that the supplemental stimuli will differ in meaningful and significant ways from the lost intrinsic signals, such as with respect to the embedded reference frame (e.g., retinocentric vs. body-centered encodings; [62]). In this case, subjects would need to learn novel mappings between changes in motor variables (e.g., muscle activations), movement kinematics (joint rotations), and changes in the supplemental kinesthetic feedback (e.g., [63,64,65]). While people can learn visuomotor rotations after some tens of movements [66,67] and they can learn truly novel visuomotor mappings for planar target capture tasks after several hundred movement attempts (c.f., [64,65]), we expect that full integration of vibrotactile feedback into the ongoing control of reaching and stabilization will be a skill that will likely require hours of practice to fully acquire. While it is possible that sensory substitution might have played some role, especially for subject S03 as described below, the fourth possibility, “cognitive processing”, most likely conferred immediate utility to supplemental vibrotactile kinesthetic feedback for stabilization (all three subjects) and reaching (subject S02) with the contralesional arm.

“Cognitive processing”, refers to the development of conscious associations and rules governing a voluntary response to the supplemental vibrotactile kinesthetic stimuli. The same set-up procedure that allowed us to verify that subjects could perceive unique vibrotactile stimuli at each of the four stimulus locations also allowed the subjects to learn how intensity of vibration at each location mapped onto hand deviations from a desired location. At the end of the set-up procedure, each subject was required to place the cursor at the center of the screen, and then to move away from that location in each cardinal direction so as to begin to learn the mapping between changes in hand position and changes in vibrotactile stimulation. Indeed, at the end of reach testing, S02 described how he implemented a specific cognitive strategy to independently and sequentially resolve performance errors along each cardinal axis of the vibrotactile interface. First he moved in the left/right direction so that he could attend to one pair of vibrators, and then he moved in the anterior/posterior direction so that he could attend to the other set of vibrators. This “decomposition strategy” for minimizing target capture errors was adopted also by healthy individuals in a prior study using the same vibrotactile display [22].

S03 also described strategic cognitive strategies for solving the reaching and stabilization tasks. Specifically, S03 reported that performing the reaching and stabilization tasks imposed a cognitive load like a dual task. While S03 indicated that she was able to feel the vibrations at the beginning of the study, and while she could correctly describe what the vibrations meant, she had difficulty transferring the information from perception to action: “as if my brain does ‘feel vibration’ and ‘moves arm’ separately”. However with practice, S03 solved this problem with different strategies for the two tasks. When reaching, she stated that she focused her attention on the vibrations instead of the residual sensation of movement. During stabilization by contrast, she stated that she focused her attention on the moving arm without paying as much attention to the vibrations. This outcome is remarkable because despite her low scores on the clinical NSA tests of somatosensation, two days of practice with supplemental vibrotactile kinesthetic feedback led to stabilization test trial performance that was markedly better that baseline performance without the vibrotactile stimuli. As noted by Sienko and colleagues [60], more than one of the sensory augmentation mechanisms can occur simultaneously, and we speculate that the strategic focus of S03 on residual sensations in her contralesional moving arm may have promoted mechanisms of sensory substitution and/or sensory integration. In any event, these promising pilot results motivate future controlled studies designed to quantify the potential contributions of sensory integration, sensory substitution, and cognitive processing to the benefits of supplemental vibrotactile kinesthetic feedback that may accrue as subjects practice reaching and stabilizing with their contralesional arm after stroke.

### 4.2. Limitations and Future Directions

This proof-of-concept case series study had several notable limitations. First, the small number of research participants and differences in testing protocol across participants limit the possibility to draw general conclusions beyond observations of objective utility and perceived usefulness. For example, whereas S01 and S03 were able to use error feedback to improve stabilization performance, S02 performed better when using state feedback. State feedback differs from error feedback in the stabilization task because state feedback includes additional hand velocity information that provides a “leading indicator” of which direction the hand will continue to move in the short term. While velocity information might be useful from a control theoretic standpoint, it might also be more confusing to interpret than error feedback, and indeed, all three subjects reported that error feedback was easier than state feedback to understand and use. Future studies should recruit a larger number of stroke survivors into a controlled study designed to assess whether the patterns of utility and perceived usability obtained in the present study may apply to stroke survivors more generally.

Second, all three subjects were exposed to state feedback on the first testing day and error feedback on the second (and later) testing day(s). We acknowledge that this ordering could have biased subjective assessments and objective performance toward error feedback because subjects had more practice on the tasks overall by the time they were introduced to error feedback. Future studies comparing the utility and usability of state and error feedback encodings should counterbalance the presentation sequence across subjects to mitigate potential order effects.

Third, the current study was structured to test whether a convenience sample of chronic stroke survivors could find supplemental vibrotactile kinesthetic feedback useful and usable for enhancing kinematic performance of reaching and stabilizing behaviors performed with the contralesional arm. We only assessed the subjective experience using free responses prompted by open-ended questions. Future studies should supplement verbal reporting with formal tools such as the System Usability Scale (SUS; [68]), which is a quick, reliable, 10-item questionnaire for measuring the usability of a system, and the Quebec User Evaluation of Satisfaction with Assistive Technology (QUEST; [69]), which was designed to evaluate user satisfaction with a wide range of assistive technologies.

A fourth limitation derives from the fact that subjects in the current study used the vibrotactile system for only about 1 h per day, for at most 3 days. We speculate that the system may confer greatest utility if it is worn continuously as a real-time sensorimotor control aid in order to provide continuous benefit through sensory substitution and/or augmentation. Moving forward, it will be important to understand the extent to which perceived vibrotactile sensitivity, alertness, and/or general body awareness (see Section 3.1.2.) adapt over time, and whether this may impact the functional utility of the wearable technology. Future studies should examine potential direct and indirect benefits that might accrue due to long term use of the wearable technology, both over the course of a full day of use and over the course of weeks and months of practice.

A fifth limitation is that the study was not designed to elucidate potential mechanisms by which supplemental vibrotactile kinesthetic feedback may enhance kinematic performance. Future studies should include additional test conditions and several days of training to determine the extent to which observed benefits of supplemental feedback may be due to sensory integration, sensory substitution, and/or cognitive processes. For example, by including *V−T−* baseline blocks of trials before and after each day of *V_KR_T+* training on a given encoding scheme, it would be possible to determine the time course and extent to which supplemental kinesthetic feedback training leads to improved performance through a beneficial re-weighting of residual task-relevant somatosensory signals (pathways). By including appropriate dual-task testing conditions and extended periods of training, it would be possible to determine the time course and extent to which integration of supplemental feedback into the planning and ongoing control of movement becomes automatic (i.e., less dependent on strategic cognitive transformations from perception to action dependent on attentional resources; c.f., [70]). Reducing cognitive load would make the proposed technology easier to use and more practical for applications where users must also be responsive to the external and uncontrolled environment.

The results presented here in a small cohort of participants suggest that many stroke survivors can perceive vibrotactile stimulation applied to the ipsilesional arm, can come to understand how to interpret it to control goal-directed behaviors performed with the contralesional arm, and that performance improvements in reaching are seen across multi-day practice sessions. Future multi-session learning studies will need to be conducted to extend these results to a larger cohort of stroke survivors, to minimize potential order effects, and to allow participants the time to develop the skill needed to autonomously integrate supplemental kinesthetic feedback into ongoing control of the arm and hand while performing real-world tasks in unstructured environments. We are encouraged in this goal because all three stroke survivors enrolled in this study found the vibrotactile feedback to be a positive experience, and some even reported secondary benefits in terms of alertness or body awareness. Such outcomes, if replicated in a larger cohort of stroke survivors, would support and encourage the use of vibrotactile feedback devices moving forward.

## 5. Conclusions

Many activities of daily living, such as reaching for a cup of water or using a spoon to eat soup, require goal-directed movements and stabilization of hand-held objects. Stroke commonly impairs aspects of somatosensation that contribute to the effective control of limb movement and stabilization. Such deficits can contribute to patterns of behavioral change called “learned non-use”. In this proof-of-concept pilot study, we sought to assess the feasibility and immediate effects of using two forms of supplemental kinesthetic feedback to enhance the accuracy and precision of goal-directed stabilization and reaching tasks performed without visual feedback by survivors of stroke in the chronic stage of recovery. Movement kinematics were encoded by a vibrotactile interface in two ways: state feedback—an optimal combination of hand position and velocity; and error feedback—the difference between the actual hand position and its instantaneous target. All three stroke survivors improved performance in the stabilization task using at least one of the feedback encoding schemes within one or two experimental sessions. These preliminary results show that supplemental vibrotactile kinesthetic feedback can be readily interpreted and exploited to improve reaching and object stabilizing actions performed with the contralesional arm after stroke. All three subjects reported that using the technology was a positive experience, while two reported an increase in alertness or general body awareness that persisted for some time after the experimental session was over. Ultimately, this line of research seeks to precipitate changes in behavior that mitigate learned non-use after stroke by enhancing control of the contralesional limb through some combination of sensory augmentation and improved sensory integration, and, possibly, through secondary effects that promote alertness and increased body awareness.

## Figures and Tables

**Table 1:** Demographic and clinical data for the participating subjects.

Subject	Gender	Age (ys)	Type	PS	TSS (ys)	Lesion Location
S01	F	68	I	R	12.5	Left basal ganglia, internal capsule, occipital lobe
S02	M	57	I	L	1	Right basal ganglia, temporal lobe, insula
S03	F	65	H	L	16	Right occipital lobe

Abbreviations: S01–S03: Subject identifiers; F: female; M: male; I: ischemic; H: hemorrhagic; PS: paretic side; R: right; L: left; TSS: time since stroke.

**Table 2:** Clinical test results.

Subject	FMA-UE		MAS						CAHAI	NSA		Tuning Fork Test			
	A–D	H	Sh	El	Fa	Wr	Fg	Th		P	S	Contra		Ipsi	
	(0–66)	(0–12)	(0–4)	(0–4)	(0–4)	(0–4)	(0–4)	(0–4)	(0–91)	(0–3)	(0–2)	El	Wr	El	Wr
S01	57	11	1	0	0	0	0	1	80	3	2	6	6	6	6
S02	6	7	1+	1+	2	3	3	3	13	0	0	6	5.5	7	7.5
S03	42	7	1	1	1	2	1	1	24	1	0	5	6	6	6

Abbreviations: FMA-UE: Upper Extremity portion of the Fugl-Meyer Assessment; A–D: motor sections; H: sensory section; MAS: Modified Ashworth Scale; Sh: shoulder; El: elbow; Fa: forearm; Wr: Wrist; Fg: Finger; Th: thumb; CAHAI: the 13-item Chedoke Arm and Hand Activity Inventory; NSA: Nottingham Sensory Assessment; P: proprioception; S: stereognosis; Contra: contralesional arm, Ipsi: ipsilesional arm.

**Table 3:** Sequence of testing conditions in each of the three test cases.

Subject	Day: Encoding	Familiarization V+T−	Practice V_KR_T+			Baseline V−T−	Test V−T+
S01	Day 1: State	R + S	R	R		R + S	R + S
	Day 2: Error		R	R		R + S	R + S
S02	Day 1: State	R + S	R	R	R	R + S	R + S
	Day 2: Error		R	R	R		R + S
S03	Day 1: State	R + S	R			R + S	
	Day 2: Error		R	R	R		R + S
	Day 3: Error		R	R	R	R + S	R + S

The order of the blocks corresponds to the timeline in which the blocks were presented in the experimental session. Abbreviations: V+T−: concurrent visual feedback without vibrotactile feedback; R: reaching, S: stabilization; V_KR_T+: vibrotactile feedback and visual knowledge of results; V−T−: neither visual nor vibrotactile feedback; V−T+: only vibrotactile feedback; orange shading: the subject used the state feedback encoding scheme; blue shading: the subject used the error feedback encoding scheme.

## Data Availability

The data presented in this study are available on request from the corresponding author. The data are not publicly available due to patient privacy considerations (HIPPA).
